# Supplementary material for: Gene Expression Noise Produces Cell-to-Cell Heterogeneity in Eukaryotic Homologous Recombination Rate
Source: Front Genet. 2019 May 21;10:475. doi: 10.3389/fgene.2019.00475 (PMC6536703; doi:10.3389/fgene.2019.00475)
Supplement: Supplementary file 1 [file Data_Sheet_1.docx]

Supplementary Material

Gene expression noise produces cell-to-cell heterogeneity in eukaryotic homologous recombination rate

Jian Liu, Jean-Marie François and Jean-Pascal Capp*

*** Correspondence:** [capp@insa-toulouse.fr](mailto:capp@insa-toulouse.fr)


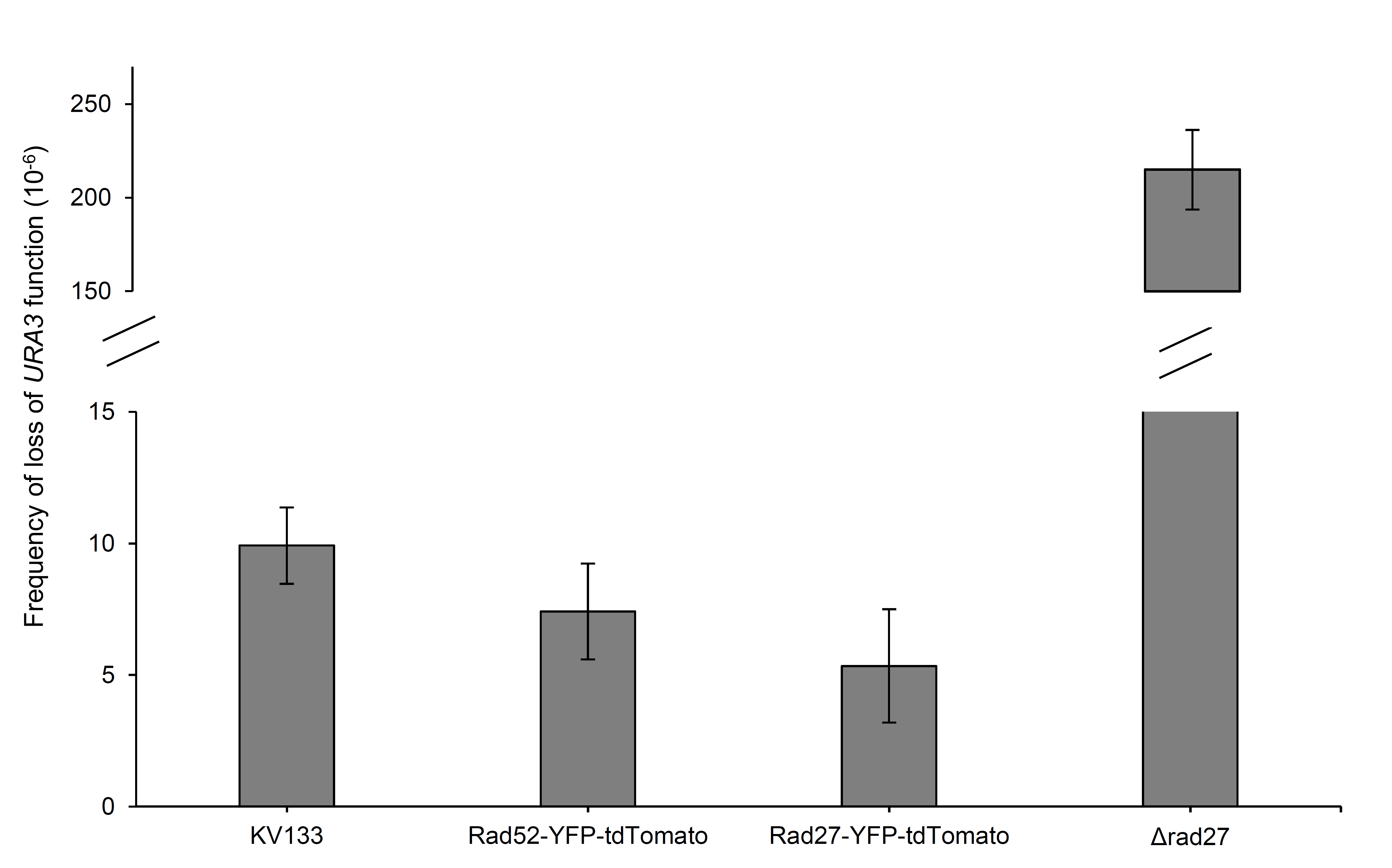


**Supplementary Figure 1.** Average spontaneous frequency of loss of URA3 function in whole populations. Results are the mean of 3 independent experiments with standard deviation.


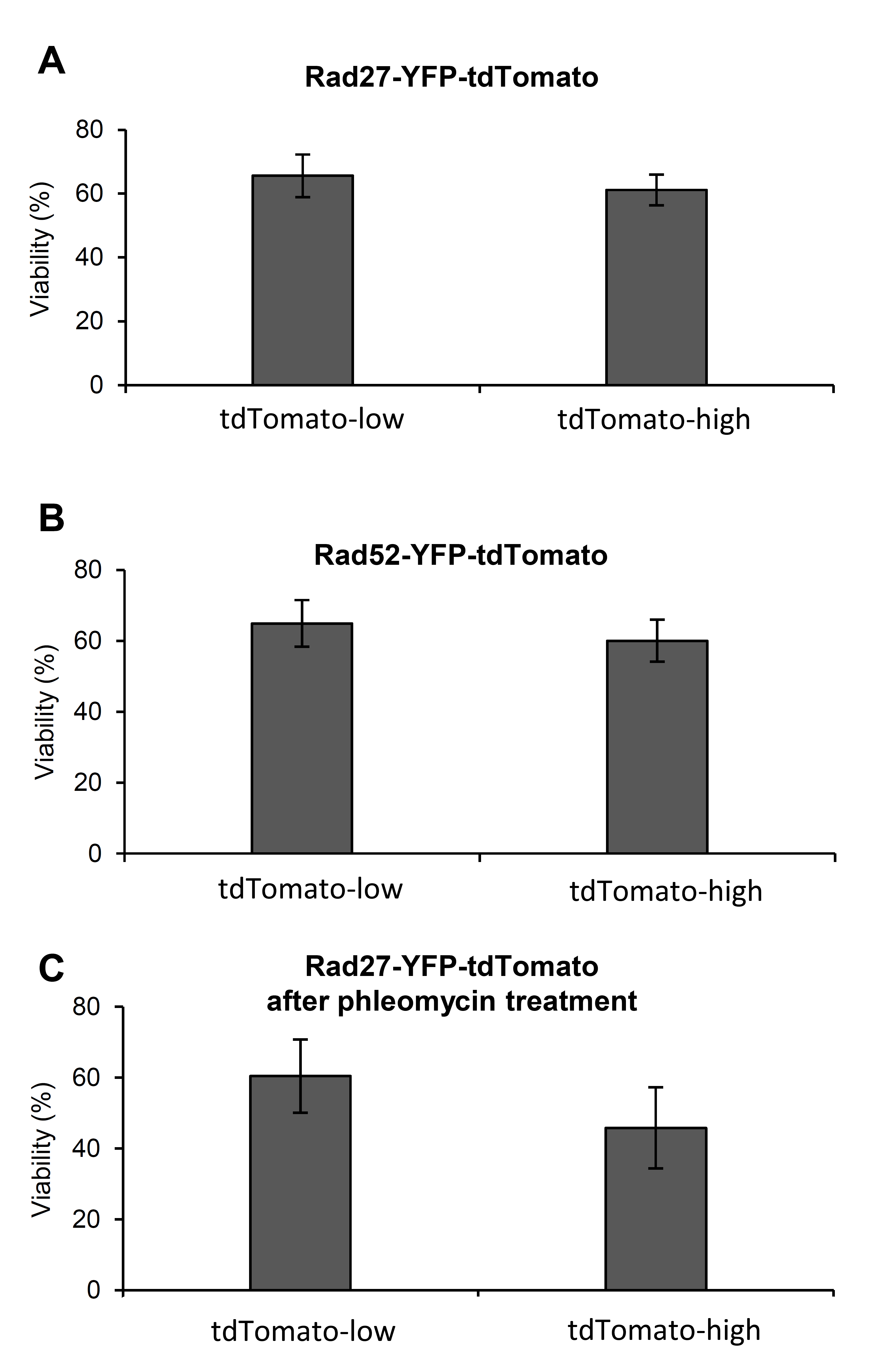


**Supplementary Figure 2.** Viability (in % of survival) in the different subpopulations used to measure frequencies of loss of URA3 function in Figure 2. Results are the mean of 3 independent experiments with standard deviation.


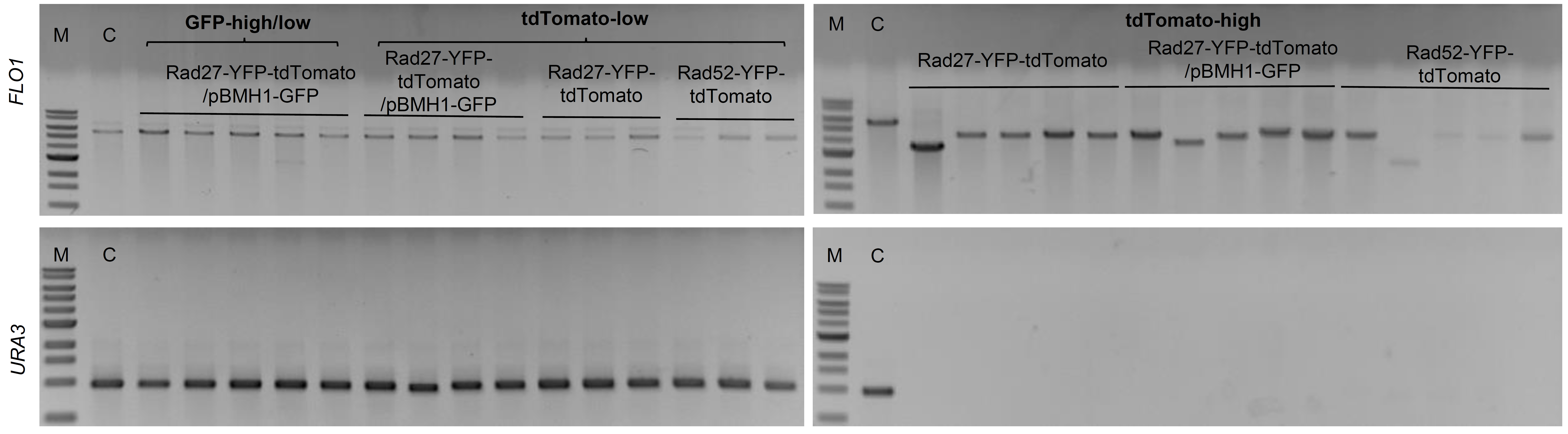


**Supplementary Figure 3**. PCR amplification of the FLO1 and URA3 alleles in different clones obtained on 5-FOA plates. Examples of PCR amplification of the new FLO1 alleles in 5-FOA resistant clones showing that their length is modified in the high-expressing subpopulations, and not in the low-expressing subpopulations compared to the control strain (C). PCR amplification of the URA3 gene in the same clones showed that it is lost by HR in the high-expressing subpopulations and still present in the low-expressing subpopulations.

**Supplementary Figure 4**. PCR amplification of the FLO5 and FLO9 alleles in different clones obtained on 5-FOA plates. Examples of PCR amplification of other loci containing tandem repeats (FLO5 and FLO9) in the FLO1 recombinant clones compared to the control strain (C).


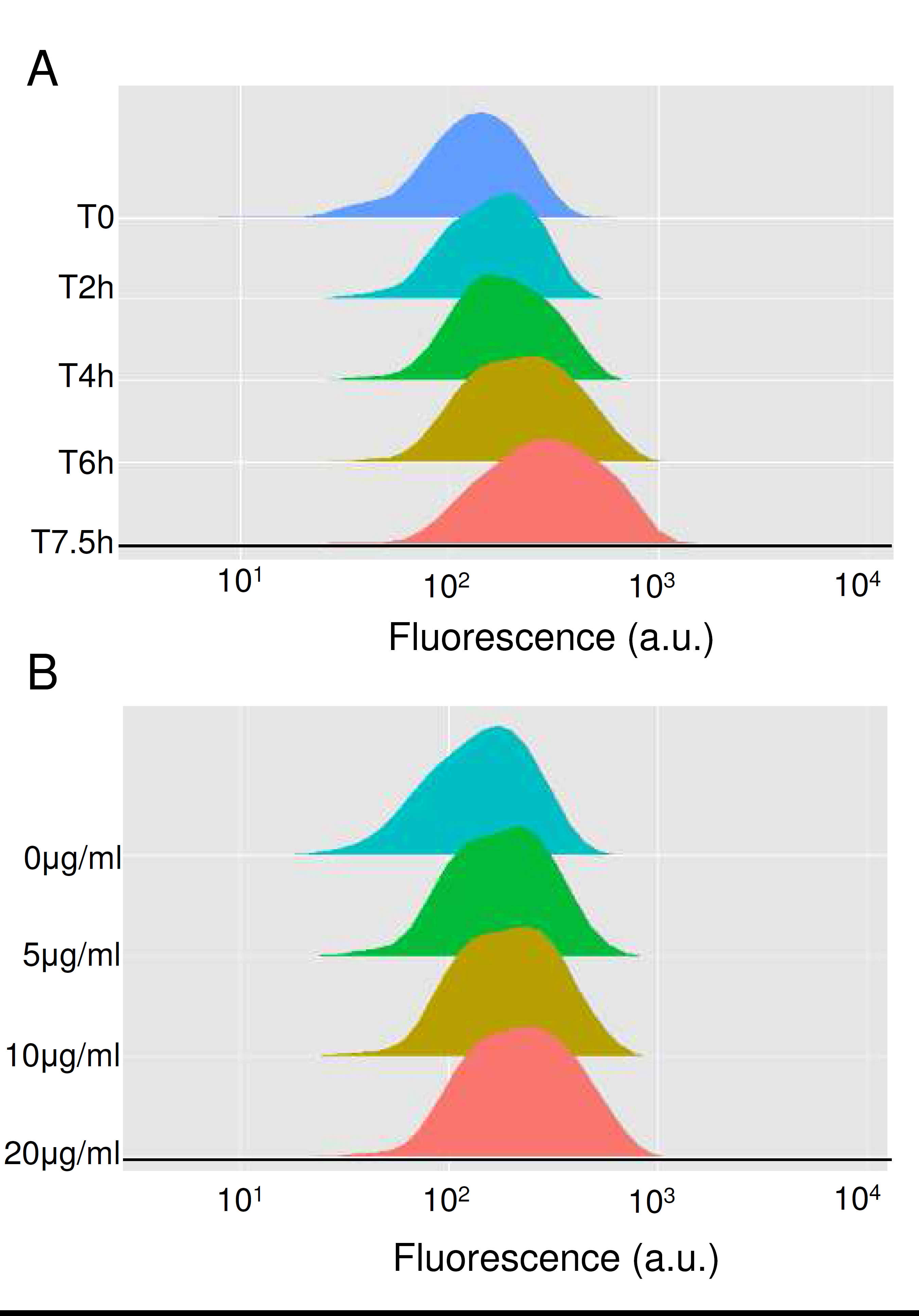


**Supplementary Figure 5**. Induction of the BMH1 promoter fused to GFP by phleomycin. Either (A) time-dependent induction in 20 µg/mL phleomycin (measurements at 0h, 2h, 4h, 6h and 7.5h) or (B) dose-dependent induction after 6h in 0, 5, 10 or 20 µg/mL phleomycin were performed.


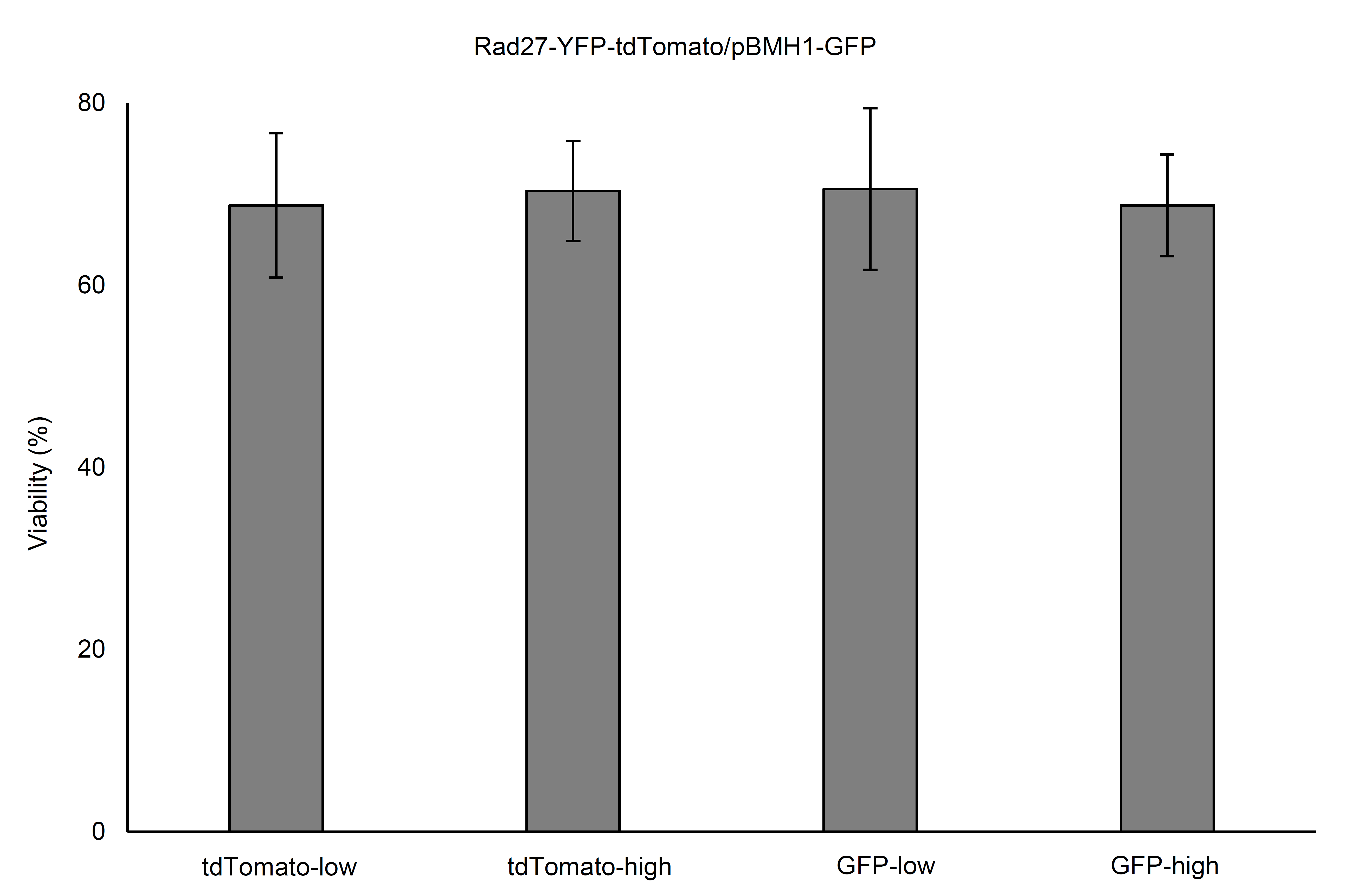


**Supplementary Figure 6**. Viability (in % of survival) in the different subpopulations used to measure frequencies of loss of URA3 function in Figure 4. Results are the mean of 3 independent experiments with standard deviation.

**Supplementary Table 1**. Raw data of the recombination rate analyses.

See enclosed .xlsx file


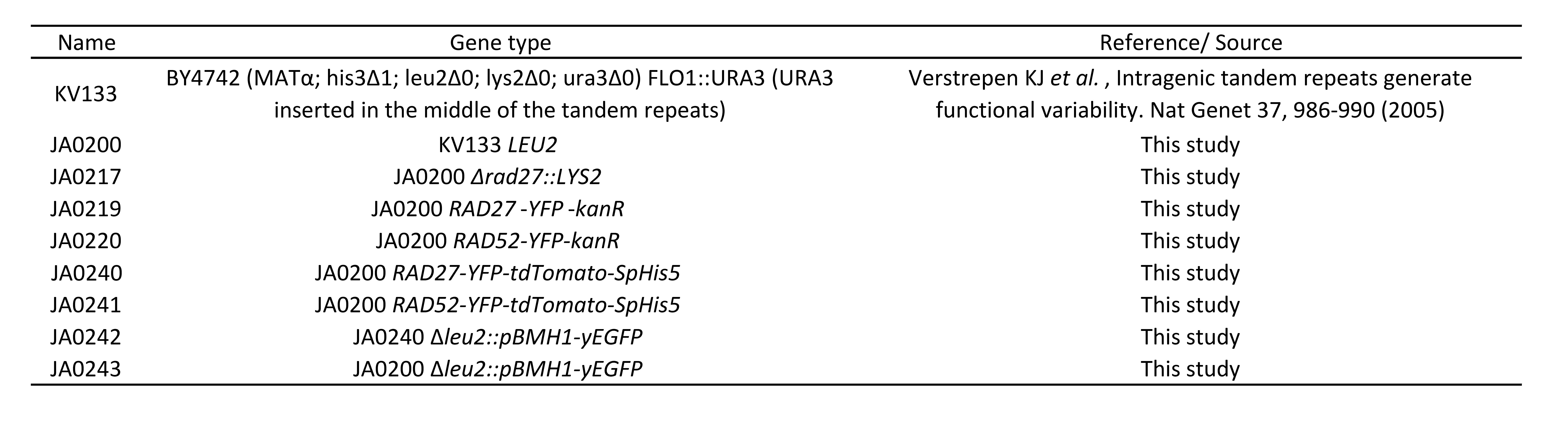


**Supplementary Table 2**. List of the strains used in this study


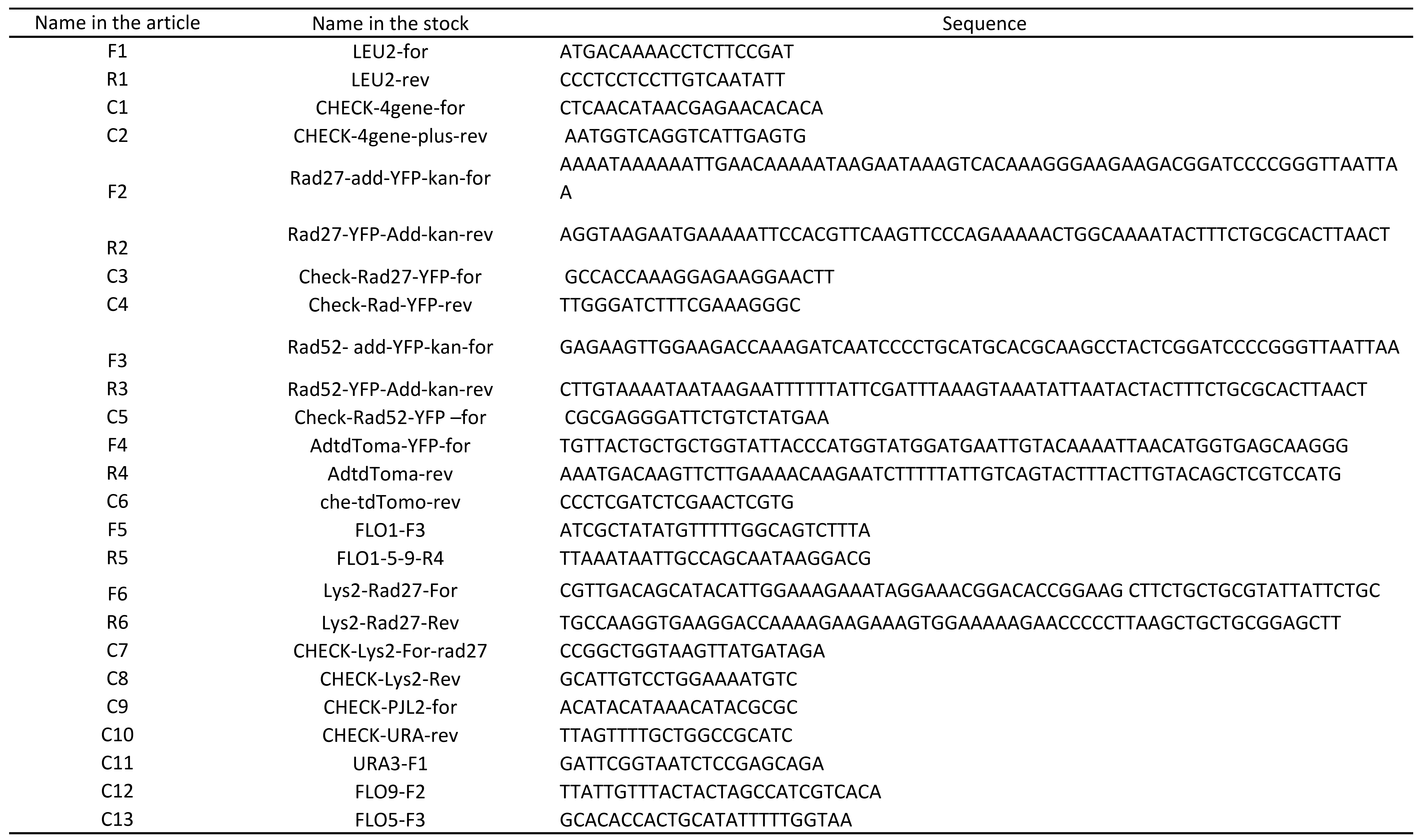


**Supplementary Table 3**. List of the primers used in this study
